# CRP Serum Levels Are Associated with High Cardiometabolic Risk and Clinical Disease Activity in Systemic Lupus Erythematosus Patients

**DOI:** 10.3390/jcm11071849

**Published:** 2022-03-26

**Authors:** Karen Pesqueda-Cendejas, Isela Parra-Rojas, Paulina E. Mora-García, Margarita Montoya-Buelna, Adolfo I. Ruiz-Ballesteros, Mónica R. Meza-Meza, Bertha Campos-López, Melissa Rivera-Escoto, Barbara Vizmanos-Lamotte, Sergio Cerpa-Cruz, Ulises de la Cruz-Mosso

**Affiliations:** 1 Proyecto Inmunonutrición y Genómica Nutricional en las Enfermedades Autoinmunes, Centro Universitario de Ciencias de la Salud, Universidad de Guadalajara, Guadalajara 44340, Mexico; karen.pesqueda20@gmail.com (K.P.-C.); iprojas@yahoo.com (I.P.-R.); moragarciapaulinaesmeralda@gmail.com (P.E.M.-G.); margaritamontoyabuelna@gmail.com (M.M.-B.); adolfo.ruba@gmail.com (A.I.R.-B.); monimez28@hotmail.com (M.R.M.-M.); bertha.campos@live.com (B.C.-L.); melissa.rivera.e@hotmail.com (M.R.-E.); bvizmanos@yahoo.com.mx (B.V.-L.); 2 Instituto de Nutrigenética y Nutrigenómica Traslacional, Centro Universitario de Ciencias de la Salud, Universidad de Guadalajara, Guadalajara 44340, Mexico; 3 Laboratorio de Investigación en Obesidad y Diabetes, Facultad de Ciencias Químico-Biológicas, Universidad Autónoma de Guerrero, Chilpancingo de los Bravo 39087, Mexico; 4 Laboratorio de Inmunología, Departamento de Fisiología, Centro Universitario de Ciencias de la Salud, Universidad de Guadalajara, Guadalajara 44340, Mexico; 5 Departamento de Reumatología, O.P.D. Hospital Civil de Guadalajara Fray Antonio Alcalde, Guadalajara 44280, Mexico; sacer04@prodigy.net.mx

**Keywords:** C-reactive protein, cardiovascular risk, systemic lupus erythematosus, clinical activity, lipid profile, body composition

## Abstract

Systemic lupus erythematosus (SLE) patients have a higher frequency of cardiovascular risk factors such as high C-reactive protein (CRP) levels than the general population. CRP is considered a cardiovascular disease marker that could be related to SLE clinical disease activity. This study aimed to assess the association between CRP with cardiometabolic risk and clinical disease activity in SLE patients. A comparative cross-sectional study was conducted in 176 female SLE patients and 175 control subjects (CS) with median ages of 38 and 33 years, respectively; SLE patients were classified by the 1997 SLE-ACR criteria, and the clinical disease activity by the Mexican-SLEDAI (Mex-SLEDAI). CRP and lipid profile (triglycerides, cholesterol, HDL-C, and LDL-C) were quantified by turbidimetry and colorimetric-enzymatic assays, respectively. SLE patients had higher CRP levels than CS (SLE: 5 mg/L vs. CS = 1.1 mg/L; *p* < 0.001). In SLE patients, CRP levels ≥ 3 mg/L were associated with a higher risk of cardiometabolic risk status assessed by LAP index (OR = 3.01; IC: 1.04–8.7; *p* = 0.04), triglycerides/HDL-C index (OR = 5.2; IC: 2.1–12.8; *p* < 0.001), Kannel index (OR = 3.1; IC: 1.1–8.1; *p* = 0.03), Castelli index (OR = 6.6; IC: 2.5–17.8; *p* < 0.001), and high clinical disease activity (OR = 2.5: IC: 1.03–6.2; *p* = 0.04; and β coefficient = 5.8; IC: 2.5–9.4; R^2^ = 0.15; *p* = 0.001). In conclusion, high CRP levels were associated with high cardiometabolic risk and clinical disease activity in SLE patients.

## 1. Introduction

Systemic lupus erythematosus (SLE) is a prototypical chronic autoimmune inflammatory disease characterized by the production of autoantibodies against self-antigens such as deoxyribonucleic acid (DNA), proteins, and nucleosomes [1], where genetic, environmental, and hormonal factors are involved. However, the exact mechanism of its pathogenesis remains unknown [2]. The breakdown of self-tolerance and the altered innate responses against self-antigen induce antibody production, leading to the deposition of immune complexes in tissues and complement activation. These aberrant mechanisms are considered to be responsible for the clinical manifestations in SLE patients [2]. 

In SLE, mortality presents a bimodal pattern, with an initial peak due to clinical disease activity and a late peak attributable to the development of cardiovascular disorders [3]. Cardiovascular disease (CVD) is one of the major causes of morbidity and mortality in SLE; it is related to traditional CVD risk factors such as dyslipidemia, obesity, and smoking. Additionally, non-traditional risk factors derived from the SLE pathophysiology, such as the glucocorticoid treatment, and inflammatory mediators such as type 1 interferons (IFN), tumor necrosis factor-alpha (TNF-α), and C-reactive protein (CRP) are involved in CVD development [4,5,6].

CRP, a liver-derived acute-phase protein produced by hepatocytes mainly in response to the inflammatory cytokine interleukin 6 (IL-6), is considered a sensitive biomarker of bacterial infections, cardiovascular events, and inflammatory conditions [7]. The physiological functions of CRP are to increase phagocytosis and activate the classical pathway of the complement, which supports complex immune clearance. In healthy individuals, CRP circulates at low concentrations; its levels increase considerably in response to infection, tissue injury, and inflammation [8]. CRP has been suggested as a powerful predictor of CVD independent of other factors in the general population. It has a relevant role in atherosclerotic plaque formation, maturation, destabilization, and rupture; therefore, CRP is described as a predictor for arterial thrombotic events and tissue damage [7,8]. Recently, CRP apheresis has been suggested as an alternative translational therapy to reduce CRP levels and tissue damage, using a phosphocholine-derivative matrix as a ligand for CRP, which could be useful to selectively deplete CRP from blood plasma in patients recovering from acute myocardial infarction [9], and other inflammatory conditions such as SLE. 

Concerning the pathogenic role of CRP in autoimmune diseases, it has been reported that SLE patients could have higher CRP serum levels compared to healthy controls [10], and the CRP serum levels correlate with traditional cardiovascular risk factors such as dyslipidemia, obesity, and glucose disturbances, which could be associated with a negative impact on robust outcomes such as damage, disease activity, and survival in SLE [11]. 

However, CRP’s role in active SLE is still complex and controversial, some studies have reported that CRP levels are normal or modestly elevated in active SLE, and that there is no relationship between CRP levels and clinical disease activity [12,13]. Nevertheless, it is widely described that in active SLE patients there is an increase in inflammatory cytokines such as IL-6, which could directly drive the CRP serum levels, suggesting a potential relationship between the increase in IL-6 in the active SLE and higher CRP serum levels [7]. Previous studies have described that CRP serum levels correlate with clinical disease activity when evaluated by the SLEDAI-2K index, where it is proposed that CRP levels could reflect the clinical disease activity in SLE [11]. Notably, SLE patients have high cardiometabolic risk, which has been related to a high clinical disease activity [14]. Therefore, based on these previous findings, our study aimed to assess the association of CRP levels with cardiometabolic risk and clinical disease activity in SLE patients. 

## 2. Materials and Methods

### 2.1. Subjects

We performed a comparative cross-sectional study on 176 female SLE patients from an unrelated Mexican Mestizo population, classified according to the 1997 American College of Rheumatology (ACR) criteria for SLE [15], recruited in 2017–2020 from the Rheumatology Department of the Hospital Civil Fray Antonio Alcalde, Guadalajara, Jalisco, Mexico.

The Mexican-Systemic Lupus Erythematosus-Disease Activity Index (Mex-SLEDAI) was used to evaluate clinical disease activity [16]. The SLE participants were without a previous diagnosis of CVD, no recent infections, trauma, surgery, pregnancy, or other autoimmune systemic conditions not related to the SLE.

The control group was 175 women recruited from the same geographical area. These control subjects (CS) did not have any recent infections, trauma, surgery, pregnancy, or autoimmune conditions; also, they did not refer family history of autoimmune diseases. 

### 2.2. Ethical Considerations

This study was approved by the Research Ethical Committee of the University of Guadalajara (CI-05018 CUCS-UdeG), based on the international ethical guidelines. All the participants gave written informed consent for their participation. 

### 2.3. Anthropometric Evaluation and Their Definitions

The anthropometric evaluation involved measurements of weight, fat mass, and muscle mass, which were determined in the morning through the bioimpedance analysis prediction method (TANITA^®^ Ironman™ body composition Monitor BC-549, Arlington Heights, IL, USA), and height was measured to the nearest 0.1 cm using a stadiometer (Seca, Hamburg, Germany). Waist and hip circumferences were measured twice using a flexible metal tape with an accuracy of ±0.1 cm (Lufkin^®^ executive thinline W606ME, Missouri City, TX, USA), with the subject standing with feet together and arms crossed. Waist circumference was measured at the midpoint between the costal margin and iliac crest in the mid-axillary line in standing position at the end of a gentle expiration, and a hip circumference measurement was taken around the widest portion of the buttocks [17].

From these measurements, body mass index (BMI) was calculated (BMI = weight, kg/height^2^, m^2^) according to the NOM-043-SSA2-2012-MEX based on World Health Organization (WHO) criteria [18]. The waist to hip ratio (WHR) was calculated (WHR = waist circumference, cm /hip circumference, cm) and classified to assess the distribution of abdominal fat in gynecoid (<0.85) or android (≥0.85); waist circumference was classified as high risk (≥80 cm) or low risk (<80 cm) for metabolic complications; the Waist to height ratio (WHtR) was calculated (WHtR = waist, cm/height, cm) and a score ≥ 0.5 was classified as a risk for metabolic abnormalities, according to the WHO cutoff values [18,19,20]. 

### 2.4. Hs-CRP Quantification

The quantification of CRP was determined using a high-sensitivity turbidimetric latex immunoassay with the high sensitivity CRP (hs-CRP) kit (COD 31927, BioSystems^®^, Barcelona, Spain); the detection limit of the assay was 0.06 mg/L, and the measurement interval was 0.06–15 mg/L.

### 2.5. Biochemical Measurements

Blood serum was taken from the participants after an overnight fast of 12 h; glucose and lipid profiles (triglycerides, total cholesterol, HDL-C, and LDL-C) were determined using a piece of semi-automated equipment (Mindray-BS-240 Clinical Chemistry Analyzer, Shenzhen, China) and colorimetric enzymatic assays (BioSystems^®^ kits, Barcelona, Spain).

### 2.6. Biochemical and Cardiometabolic Criteria Definitions

To evaluate the CVD risk, we applied cut-points for CRP levels at low risk (<1.0 mg/L), average risk (≥1.0 to <3.0 mg/L), and high risk (≥3.0 mg/L) based on the criteria of the Centers for Disease Control and Prevention and the American Heart Association [21]. Cardiometabolic indexes were calculated and interpreted according to formulas described in detail in a previous study by Campos-López et al. [14]: (a) Castelli index classified as low (<4.5), moderate (≥4.5 to <7.0) and high (≥7.0) CVD risk; (b) Kannel index classified as low (<3) and high (≥3) CVD risk; (c) TG/HDL-C ratio classified as elevated a score ≥3 [22]; (d) cardiometabolic index (CMI score) [20] classified by tertiles (T): T1st (minimum value to <0.6069) of low CVD risk, T2nd (≥0.6069 to <1.188) and T3rd (≥1.188 to maximum value), these last two considered as medium and high CVD risk, respectively; (e) lipid accumulation products (LAP score) [23] were classified as: (T): T1st (minimum value to <10.74) of low CVD risk, T2nd (≥10.74 to <31.06) and T3rd (≥31.06 to maximum value), these last two were considered as medium and high CVD risk, respectively. 

### 2.7. Statistical Analysis

The statistical analyses were performed with the software STATA v 9.2 (College Station, TX, USA) and GraphPad Prism v 5.0 (San Diego, CA, USA). The statistical power was evaluated according to the calculation of sample size, performed with an estimated error margin of 2% with a confidence degree of 95%, and expected prevalence of 60% for dyslipidemia after three years of disease evolution time in SLE patients reported in previous studies [7,8]. The normal variable distribution was assessed by the Shapiro–Wilk test. For descriptive analysis, categorical variables are expressed as frequencies; continuous variables with nonparametric variables are expressed as medians and percentiles 5th–95th.

For inferential analysis, the Fisher χ^2^ test was used to compare proportions. Mann–Whitney U test was used for nonparametric quantitative determinations of two groups, and for nonparametric quantitative determinations of three groups, Kruskal–Wallis test was applied. The discriminative capacity of CRP to differentiate between SLE patients vs. CS, and active vs. inactive SLE patients was calculated using a receiver operator characteristic (ROC) curve, and the area under the curve (AUC) from the receiver operating characteristic was calculated. To determine the correlations between CRP with cardiometabolic variables and clinical disease activity, we used Spearman correlation tests. The associations of CRP with cardiometabolic indexes and the clinical disease activity were determined by logistic regression models to estimate odds ratios, and by linear regression models to estimate β coefficients, using adjusted models. The differences were considered significant at a *p* value < 0.05.

## 3. Results

### 3.1. General Characteristics in SLE Patients and CS

A total of 176 female SLE patients with a median age of 38 years were evaluated. They presented a median of clinical disease activity of 0 (remission), 44% were active patients, and 56% were in remission; 33% of the patients had renal activity, and the median of disease duration was of 7 years. As a reference control group representative of the same population, a total of 175 CS women with a median age of 33 years were evaluated. SLE patients had higher weights (SLE = 67 vs. CS = 61.2 kg; *p* < 0.001) than CS and presented a circumference waist > 80 cm (SLE = 84 vs. CS = 76.7 cm; *p* < 0.001) classified as cardiovascular risk; additionally, SLE patients had a BMI > 25 kg/m^2^ classified as overweight (SLE = 26.9 vs. 23.6 kg/m^2^; *p* < 0.001), while CS had an adequate weight according to BMI. SLE patients, in addition, had higher WHR scores (SLE = 0.52 vs. CS = 0.47; *p* < 0.001) than CS. Regarding biochemical variables, SLE patients had higher levels of triglycerides (SLE = 117.2 vs. CS = 76 mg/dL); *p* < 0.001), and lower levels of HDL-C (SLE = 33.7 vs. CS = 50.9 mg/dL; *p* < 0.001), regarding the cardiometabolic indexes. SLE patients presented a higher score with regard to the Castelli atherogenic index (SLE = 4.8 vs. CS = 3.3; *p* < 0.001), Kannel index (SLE = 2.4 vs. CS = 1.8; *p* < 0.001), triglycerides/HDL-C ratio (SLE = 3.6 vs. CS = 1.4; *p* < 0.001), CMI score (SLE = 1.19 vs. CS = 0.7; *p* < 0.001), and LAP score (SLE = 29 vs. CS = 15; *p* < 0.001) than CS (Table 1). Concerning the SLE treatment, 52.5% of the patients received prednisone treatment with a median dose of 10 mg/day, 46% used chloroquine (CQ) with a median dose of 150 mg/day, and 30.5% hydroxychloroquine (HCQ) with a median dose of 200 mg/day; additionally, 32% were in treatment with antihypertensives (Table 1).

### 3.2. CRP Levels and Cardiovascular Risk in Active and Inactive SLE

**Figure 1:**
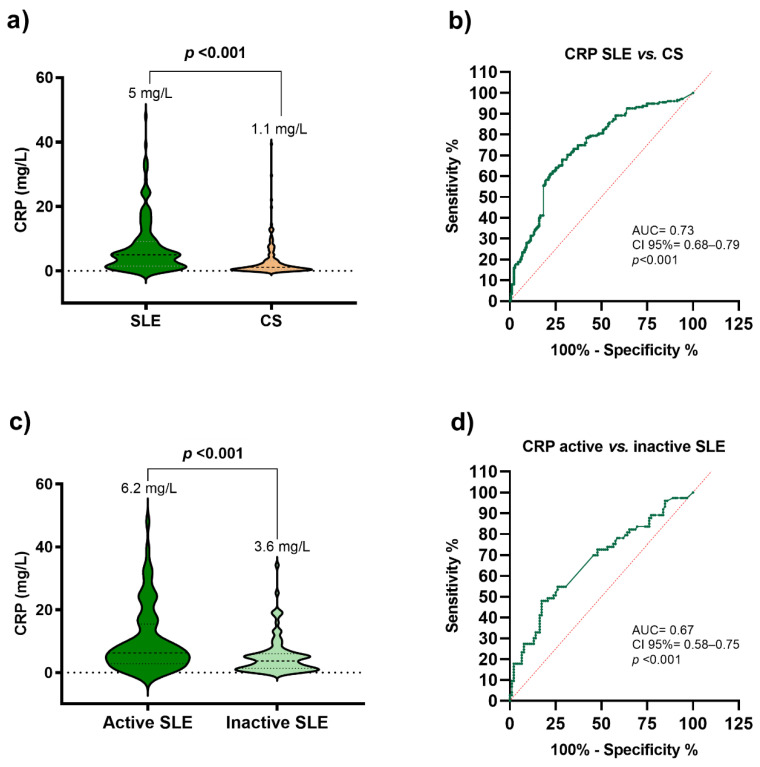
**Serum CRP levels were stratified by study groups.** (**a**) Serum CRP levels in SLE patients and CS. Data presented as median; *p* value U Mann–Whitney test. SLE: systemic lupus erythematosus patients; CS: control subjects; CRP: C-reactive protein. (**b**) Discriminatory receiver operating characteristic (ROC) curve between SLE patients vs. CS. AUC = area under the curve. 95% CI = 95% confidence interval. (**c**) Serum CRP levels stratified by clinical disease activity in SLE patients. Clinical inactivity: Mex-SLEDAI < 2; Clinical activity: Mex-SLEDAI ≥ 2. Data provided in median; *p* value U Mann–Whitney test. (**d**) Discriminatory receiver operating characteristic (ROC) curve between inactive vs. active SLE patients.

Concerning the CRP levels, SLE patients showed higher levels than the CS group (SLE = 5 vs. CS = 1.1 mg/L; *p* ≤ 0.001) (Figure 1a); then, we determined the CRP capacity to discriminate between SLE patients and CS using ROC curves. Based on these results, the CRP levels have a high capacity of discrimination with an AUC of 0.73 (CI: 0.68–0.79; *p* < 0.001) (Figure 1b). When comparing CRP levels between active SLE and inactive SLE, we observed that active SLE patients have higher CRP levels than inactive SLE (active SLE = 6.2 vs. inactive SLE = 3.6 mg/L; *p* < 0.001) (Figure 1c). The CRP levels showed a moderate capacity to discriminate between active and inactive SLE with an AUC of 0.67 (CI: 0.58–0.75; *p* < 0.001) (Figure 1d). 

### 3.3. Biochemical and Cardiometabolic Status and CRP in SLE Patients and CS

To compare the biochemical and cardiometabolic statuses according to the CVD risk by the criteria from the center for disease control and prevention and the American Heart Association to CRP serum levels, both SLE patients and CS were stratified according to CRP levels as low CVD risk (<1 mg/dL), average CVD risk (≥1 to <3 mg/dL) and high CVD risk (≥3 mg/L). SLE patients with high CVD risk showed higher triglycerides levels (*p* < 0.001) and lower HDL-C levels (*p* < 0.001) than patients with low and average CVD risk; additionally, it was observed that SLE patients with levels of CRP ≥ 3 mg/L (high CVD risk) presented higher scores with regard to the Castelli index (6.5; *p* < 0.001), Kannel index (2.5; *p* < 0.001), triglycerides/HDL-C ratio (4.4; *p* < 0.001), CMI (1.4; *p* = 0.03) and LAP score (41.7; *p* = 0.02) (Table 2). SLE patients with levels of CRP ≥ 3 mg/L also presented a higher frequency for high CVD risk according to the Castelli index (48%; *p* < 0.001), Kannel index (40.4%; *p* = 0.001) and triglycerides/HDL-C ratio (71%; *p* < 0.001). This pattern was also observed in the CS group, where CRP levels ≥ 3 mg/L showed a worse lipid profile and a higher score of the cardiometabolic indexes than subjects with CRP levels < 3 mg/L (Table 2).

Additionally, we stratified the complement C3 and C4 serum levels according to cardiovascular risk by CRP, and we did not observe significant differences in C3 according to CRP levels, but we found a tendency for lower C4 levels in SLE patients with average (≥1 mg/L) and high cardiovascular risk (≥3 mg/L) compared to SLE patients with low risk (<1 mg/L) (CRP low risk: 25.2 mg/dL; average risk: 12.5 mg/dL; high risk: 15.5 mg/dL; *p* = 0.06). Regarding the SLE treatment, patients with CQ treatment had higher CRP serum levels than SLE patients with HCQ treatment (CQ = 5 mg/L vs. HCQ = 1.7 mg/L; *p* < 0.001). According to the classification for CVD risk by CRP, a high CVD risk was observed in SLE patients with CQ treatment vs. HCQ treatment (CQ = 77.2% vs. HCQ = 22.8%; *p* < 0.001). A similar pattern was observed in SLE patients who received prednisone treatment, presenting higher CRP serum levels (with prednisone = 5.7 mg/L vs. without prednisone = 2.6 mg/L; *p* < 0.001) and a higher frequency of high CVD risk evaluated by CRP in comparison to SLE patients without prednisone treatment (with prednisone = 64.6% vs. without prednisone = 35.4%; *p* < 0.001).

Based on the previous results, we determined the correlation between body composition and cardiometabolic status with CRP levels. In all the participants we found a significant correlation between CRP levels and all the body composition and cardiometabolic variables evaluated, except total cholesterol. In the SLE group, the CRP levels positively correlated with body composition variables such as weight (r = 0.22; *p* < 0.01), BMI (r = 0.28 *p* < 0.001), WHtR (r = 0.21; *p =* 0.03) and fat mass (r = 0.21; *p* = 0.03). In the CS group, weight (r = 0.56; *p* < 0.001), waist (r = 0.60; *p* < 0.001), BMI (r = 0.63; *p* < 0.001), WHR score (r = 0.45; *p* < 0.001), WHtR score (r = 0.59; *p* < 0.001) and fat mass (r = 65; *p* < 0.001) correlated positively, while muscle mass (r = −0.61; *p* < 0.001), and body water (r = −0.65; *p* < 0.001) were negatively correlated with CRP levels (Table 3).

Regarding cardiometabolic status, in SLE patients we observed a positive correlation between triglycerides levels (r = 0.18; *p* = 0.01), the Castelli index (r = 0.35; *p* < 0.001), the Kannel index (r = 0.23; *p* < 0.01), the triglycerides-HDL-C ratio (r = 0.38; *p* < 0.001), and CMI score (r = 0.21; *p* = 0.03), and a negative correlation between LDL-C (r = −0.24; *p* = 0.001), and HDL-C (r = −0.37; *p* < 0.001) with CRP levels. Moderate correlations were observed in the CS group between CRP levels and the cardiometabolic variables. Regarding SLE patients, the CRP levels correlated positively with the Mex-SLEDAI score (r = 0.22; *p* < 0.01) and with disease duration (r = 0.20; *p* < 0.01) (Table 3).

### 3.4. Association of CRP Levels with Cardiometabolic Variables and Clinical Disease Activity

**Figure 2:**
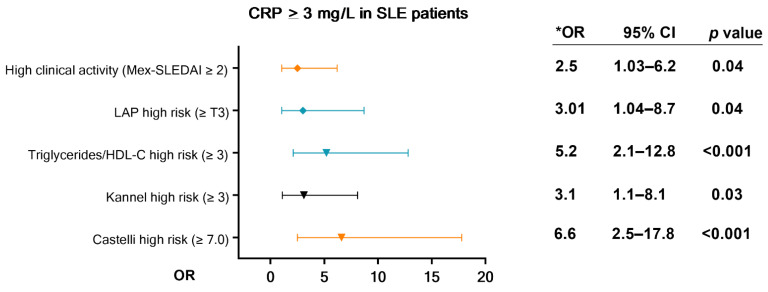
**Association of the CRP levels (≥3 mg/L) with clinical activity and cardiometabolic variables in SLE.** Castelli index: total cholesterol/HDL-C; Kannel index: LDL-C/HDL-C ratio; LAP score: (waist circumference, cm–58) (TG, mmol/L). T3: ≥31.06 to maximum value; OR: odds ratio, confidence interval 95%, *p* values < 0.05. * Reference group: SLE patients with CRP < 1 mg/L.

We analyzed the association of the high cardiovascular risk by CRP levels (≥3 mg/L) with cardiometabolic variables and clinical disease activity in SLE patients by logistic regression models. We found that SLE patients with CRP levels ≥ 3 mg/L had a significantly higher risk of presenting clinical disease activity with a Mex-SLEDAI ≥ 2 (OR = 2.5; CI = 1.03–6.2; *p* = 0.04), higher risk of having a high LAP score ≥ Tertile 3rd (OR = 3.01; CI = 1.04–8.7; *p* = 0.04), higher risk of a triglycerides/HDL-C index score ≥ 3 (OR = 5.2; IC = 2.1–12.8; *p* < 0.001), Kannel index score ≥ 3 (OR = 3.1; IC = 1.1–8.1; *p* = 0.03), and Castelli index score ≥ 7 (OR = 6.6; IC = 2.5–17.8; *p* < 0.001) in comparison with SLE patients with CRP < 1 mg/L (Figure 2).

Finally, based on the previous results, we determined in a linear regression model that the high CRP levels (≥3 mg/L) increased 6.1 points of the Mex-SLEDAI score when we adjusted the model for fat mass percentage (β coefficient = 6.1; IC = 2.8–9.1; R^2^ = 0.14; *p* <0.001). These findings suggest that the association found between CRP and the clinical activity is not influenced by fat mass. When we adjusted by other variables such as BMI, age, and fat mass percentage, the significant association remained (β coefficient = 5.8; IC = 2.5–9.4; R^2^ = 0.15; *p* = 0.001), which demonstrates that the associations found between CRP and clinical disease activity were independent of these variables that could influence the CRP serum levels.

## 4. Discussion

In this present study, we found that SLE patients had excess weight and altered waist circumference related to CVD risk. Previous studies conducted on SLE populations supported these findings; the SLE patients showed a high frequency of excess weight associated with cardiovascular disease risk factors [24,25]. SLE patients also showed higher triglycerides and lower HDL-C levels compared to CS. These findings are in accordance with the classical “lupus lipoprotein pattern”, characterized by high triglycerides and low HDL-C but unchanged LDL-C. In this pattern, there is also the presence of some autoantibodies such as anti-HDL-C and anti-lipoprotein lipase [26]; however, the lipid autoantibodies were not evaluated in this study. 

Notably, we found that SLE patients had a high cardiovascular risk determined by cardiometabolic indexes such as the Castelli index, Kannel index, triglycerides/HDL-C ratio, CMI index, and LAP index. The worse cardiometabolic status in SLE than CS can be partly explained by the high frequency of traditional risk factors such as diabetes mellitus, hypertension, central obesity, dyslipidemia, and pharmacotherapy such as glucocorticoids in these patients. Previous studies have reported rates of dyslipidemia in SLE patients ranging from 36% at the time of diagnosis to more than 60% within a three-year follow up [27]. Additionally, SLE treatment plays a relevant role in developing CVD in these patients. Prednisone use has been associated with an altered lipoprotein profile that could be a potential mechanism for the enhanced atherogenic risk in SLE. Immunological mechanisms have also been related to the development of atherosclerosis in SLE, an imbalance between endothelial damage and atheroprotective mechanisms and autoantibodies such as anti-endothelial cell (AEC) and anti-oxLDL could participate in the cardiovascular disturbances in SLE [28].

CRP is accepted as an independent risk factor for cardiovascular events in the general population [29]. It is one of the components of the Reynolds cardiovascular risk score [30], but its role in CVD and SLE is still controversial; based on previous findings, we assessed the relationship of CRP with biochemical and cardiometabolic variables. According to this, SLE patients with high cardiovascular risk assessed by CRP (≥3 mg/L) showed significantly higher triglyceride levels and lower HDL-C levels than patients at low and average risk. Patients with high CRP levels (≥3 mg/L) also presented higher scores with regard to the Castelli, Kannel, triglycerides/HDL-C, LAP, and CMI indices. Differences according to CRP levels were also observed in the CS group; these results suggest that CRP could determine cardiovascular status in an indirect way through influencing cardiometabolic variables.

Following these reported findings, we found a positive correlation between CRP levels and anthropometric variables such as weight, waist, BMI, WHR, and fat mass percentage. Previous cross-sectional studies have shown that CRP correlates with obesity indicators such as BMI, WHR, and adiposity [31,32]; some authors even suggest that the fat mass has a greater ability to classify subjects with high CRP serum levels compared with BMI and WHR [32]. The mechanism linked to CRP and fat mass could be mediated by adipose tissue, which is the main source of inflammatory cytokines; approximately 30% of total IL-6 production may arise in adipose tissue; this interleukin is the main stimulant for CRP production [33].

CRP levels are also positively correlated with biochemical variables such as triglycerides and the Castelli, Kannel, CMI, and triglycerides/HDL-C cardiometabolic indexes, and negatively correlated with HDL-C levels. Our results agree with previous studies conducted on SLE populations that reported a correlation between CRP levels and lipid profile alterations, diabetes, obesity, and BMI. They also found that triglyceride levels and the triglycerides/HDL-C ratio positively correlated with CRP levels [11]. 

The relationship between CRP and dyslipidemia could be explained by excessive lipids accumulating in the arterial wall, inducing an inflammatory response; it accelerates lipid deposition and amplifies the inflammation producing inflammatory factors such as CRP [34]. CRP can bind to LDL-C in atherosclerotic plaques, leading to complement activation, and promoting inflammation and atherosclerosis. Pan He et al. reported that CRP plays a mediator role in the relationship between dyslipidemia and coronary arterial disease [34]. On the other hand, in normal conditions HDL-C promotes reverse cholesterol transport and inhibits LDL-C oxidation; however, under inflammatory conditions characterized by high levels of inflammatory markers such as IL-6, the antioxidant and anti-atherogenic capacity of HDL-C may be lower. Additionally, the inflammatory process could alter LPL activity, resulting in the accumulation of VLDL, thereby increasing triglycerides and lowering the HDL-C serum levels [4]. However, to our knowledge, the exact mechanism through which CRP affects lipid metabolism is still unclear. 

Concerning CRP levels in SLE compared to general populations, some studies have reported no differences in CRP levels between SLE patients vs. the general population. Controversial results have been reported, where SLE patients used to have lower CRP levels than healthy subjects [35]. The mechanism suggested in other studies to alter and cause inhomogeneous levels of CRP in SLE patients are the lower CRP production through the increased production of IFN-α, which is characteristic in active SLE. This event is brought about via the enhancer binding protein (C/EBPs) and STAT3, affecting the inhibition of CRP production [35]. 

Conversely, we found that SLE patients have higher CRP levels than healthy individuals. These findings also are in accordance with other studies conducted on different SLE populations [10,12]. We also showed that CRP levels had a high capacity to discriminate between SLE and CS using ROC curves. According to this, the inconsistencies reported in the literature could be derived from the method used to determine CRP levels; conventional methods can only detect values above 3 mg/L, while CRP can detect at a level as low as 0.3 mg/L [36]. On the other hand, polymorphisms in *the CRP* gene could alter *CRP* transcription or mRNA stability depending on its location, which could increase CRP levels, according to some studies conducted on the Mexican population [37,38]. A study conducted on SLE patients from Brazil showed an association between rs1130864 *CRP* polymorphism and SLE susceptibility [39], while Enocsson et al. reported that IFN-α downregulates CRP expression, and the rs1205 *CRP* polymorphism could explain the low basal CRP and the inadequate CRP responses among active SLE patients [40]. However, it is well known that there are racial and ethnic disparities in the allele frequency distribution of *CRP* polymorphisms [41], which could explain in part the discrepancies reported in the literature about the association of *CRP* polymorphism with genetic risk and the clinical disease activity in SLE populations with different genetic backgrounds. 

The immunomodulatory role of CRP is widely described; it could exert inflammatory and anti-inflammatory activities by regulating complement activation through the binding of factor H, promoting the binding of the apoptotic materials and their clearance [36]. We showed that there are no differences in C3 complement levels according to CRP stratification, but SLE patients with average and high CVD risk by CRP levels (≥1 mg/L) tended to have lower C4 complement levels than patients with low CRP levels (<1 mg/L). A study conducted on SLE patients from China reported a negative correlation between C3 and C4 complement levels and the clinical disease activity; however, CRP serum levels were not associated with clinical disease activity [42]. 

The role of CRP in clinical disease activity in SLE is still complex and controversial; some studies reported that CRP levels are normal or only modestly elevated in active SLE and that there is no relationship between CRP levels and clinical disease activity [12,13]. In contrast, we found that patients with active SLE had higher CRP levels than inactive SLE patients; additionally, we observed a positive correlation between Mex-SLEDAI score and CRP levels when we adjusted for some variables that could influence it, such as age, fat mass, and BMI [32]. It has been reported that acute phase reactants such as CRP tend to increase with age [43]. Notably, we observed that the association between CRP levels and clinical disease activity in SLE is independent of this factor. Concerning fat mass and BMI, a previous study conducted on our SLE population reported that patients with excess weight (BMI ≥ 25 kg/m^2^) had high clinical disease activity. Additionally, a positive correlation between Mex-SLEDAI score and BMI was observed [44]. According to this, we observed that the relationship between CRP levels and clinical disease activity is also independent of the excess weight. Mok et al. reported that CRP levels were detectable in 77% of SLE patients, and CRP correlated with SLEDAI scores in SLE patients from USA [36]. Another study conducted on SLE patients from Spain also showed a significant correlation between CRP levels and the clinical disease activity assessed by the SLEDAI-2K score [11]. 

The discrepancies reported about CRP and clinical disease activity in SLE in different studies could be related to its structure; it has been reported that two structures of CRP exist: the pentameric (pCRP) and monomeric CRP (mCRP) forms. These exert different biological functions. pCRP binds and opsonizes dying cells and cell remnants, facilitating phagocytosis via Fc-receptor binding [7,45]. The mCRP form is an efficient activator of the classical complement pathway involving C1, C2, C3, and C4 complement fractions, acting as a regulator of the alternative complement pathway. mCRP is considered a more pro-inflammatory form, and could promote the differentiation of monocytes toward a pro-inflammatory M1 phenotype [46], resulting in a high secretion of inflammatory cytokines such as IL-6 and IL-1 [47]; this, in a feedback loop, induces CRP synthesis in hepatocytes at the transcriptional level through STAT3 activation [48]. Additionally, mCRP rather than pCRP has been related to cardiovascular disturbances [46]; previously, it was reported that the high cardiovascular risk determined by biochemical variables and cardiometabolic indexes in SLE patients was associated with clinical disease activity [14]. Based on this, we hypothesized that the relationship between CRP and active SLE could increase cardiovascular risk and subsequently increase the inflammatory process and clinical activity. 

In other pathologies such as cancer, the roles of pCRP and mCRP have also been described [49]. However, neither study conducted on SLE patients have reported the role of CRP according to its structure. pCRP’s structure is dependent upon the presence of calcium ions. The binding of pCRP to a damaged cell membrane, inflammatory conditions, and oxidative stress promote conformational switching from pCRP to mCRP [7]. To date, no studies have measured the conversion rate from pCRP to mCRP in SLE, and if there is a differential relationship according to CRP structure with CVD risk and clinical disease activity. In our study, we observed that the high serum levels of CRP (≥3 mg/L) were associated with high clinical activity in SLE. We also showed that SLE patients with CRP levels ≥ 3 mg/L had a high chance of being at high cardiometabolic risk, assessed by the LAP, Castelli, triglycerides/HDL-C, and Kannel indexes. Based on these results, we suggest that CRP levels could be an additional biomarker to monitor cardiometabolic risk and the clinical disease activity in SLE; however, further studies are necessary to support these findings. Additionally, CRP could be a useful target to reduce CVD risk and the clinical disease activity in SLE. In populations with cardiovascular events, the CRP apheresis technique showed to be promising for decreasing CRP levels [9], and this technique could be applied to SLE patients with CRP-mediated inflammatory conditions.

Despite its strengths, this study had limitations: first, our comparative cross-sectional design is limited by merely showing a relationship between the CRP levels, cardiometabolic status, and clinical disease activity; however, we do not suggest causality because it only provides information on a specific point in time. Second, another limitation is that some SLE patients evaluated had incomplete clinical, biochemical, and pharmacotherapy administered data that we could not retrieve from the patient’s medical records or at the time of quantifying the analytes presented in this study. Third, we were not able to assess IL-6 serum levels, which is responsible for stimulating CRP production. Finally, we only provided information about the global clinical disease activity and renal activity in our population; however, we were not able to assess the clinical activity of other organs such as the skin, joints, pleura, or central nervous system. Despite its weaknesses, the present study provides evidence of the association between high CRP levels with high cardiometabolic risk and clinical disease activity in SLE patients. 

Further prospective studies on SLE Mexican population cohorts are needed to be performed to evaluate causality in the relationship of CRP serum levels with cardiometabolic and organ-specific disease activity outcomes. Moreover, it will be necessary to evaluate the specific pathogenic role of the monomeric or pentameric CRP structures in autoimmune conditions, as well as the potential correlation between CRP, IL-6, and other pro-inflammatory cytokines in SLE. Finally, we need to determine the influence of some genetic variants in the CRP gene and the possible epigenetic interactions with genes involved in CRP synthesis.

## 5. Conclusions

The present study provides evidence of the association between high CRP levels with high cardiometabolic risk in SLE patients and the general population. Notably, in SLE patients, CRP serum levels were also associated with clinical disease activity. Therefore, CRP could be an additional biomarker to monitor cardiometabolic risk and clinical disease activity in SLE. 

## Figures and Tables

**Table 1:** General characteristics in SLE patients and CS.

Variable	SLE (*n* = 176)	CS (*n* = 175)	*p* Value
**SLE clinical features**			
Mex-SLEDAI (score) ^a^	0 (0–8)	-	-
**Mex-SLEDAI classification % (*n*)**			
Clinical disease activity (≥2) ^b^	44 (69/167)	-	-
Clinical remission (<2) ^b^	56 (86/167)	-	-
Renal activity % (*n*)	33 (32/97)	-	-
Disease duration (years) ^a^	7 (0.6–21)	-	-
**Body composition**			
Weight (kg) ^a^	67 (49.6–96.9)	61.2 (46.6–86.5)	**<0.001**
Waist (cm) ^a^	84 (67.2–104.2)	76.7 (61.5–105)	**<0.001**
BMI (kg/m^2^) ^a^	26.9 (19.5–37.5)	23.6 (18.6–34)	**<0.001**
WHR (score) ^a^	0.83 (0.73–0.93)	0.77 (0.68–0.93)	**<0.001**
WHtR (score) ^a^	0.52 (0.41–0.65)	0.47 (0.38–0.65)	**<0.001**
Muscle mass (kg) ^a^	40.7 (35.9–50.5)	39.8 (35.4–45.8)	**0.01**
Fat mass (%) ^c^	33.4 ± 8.48	32.1 ± 9	0.87
**Biochemical data**			
Glucose (mg/dL) ^a^	87.2 (71.0–133)	87.8 (75.1–118)	0.74
Triglycerides (mg/dL) ^a^	117.2 (49–242)	76 (38–198)	**˂0.001**
Total cholesterol (mg/dL) ^a^	168.8 (121–245)	169 (121–245)	0.40
HDL-C (mg/dL) ^a^	33.7 (14–64)	50.9 (32–77)	**˂0.001**
LDL-C (mg/dL) ^a^	77.5 (46–142)	95 (59–158)	**<0.001**
**Cardiometabolic indexes**			
Castelli atherogenic index (TC/HDL-C) ^a^	4.8 (2.5–13.9)	3.2 (2.17–13.8)	**˂0.001**
Kannel index (LDL-C/HDL-C) ^a^	2.4 (1.1–5.6)	1.84 (1–3.5)	**<0.001**
Triglycerides/HDL-C ratio (score) ^a^	3.6 (1–15)	1.4 (0.6–5.1)	**<0.001**
CMI (score) ^a^	1.19 (0.44–3.39)	0.7 (0.26–4.8)	**<0.001**
LAP (score) ^a^	29 (6.2–76)	15 (2.3–89)	**<0.001**
**Treatment**			
Prednisone % (*n*) ^b^	52.5 (93/177)	-	-
Prednisone dose (mg/day) ^a^	10 (5–50)	-	-
Chloroquine % (*n*) ^b^	46 (81/177)	-	-
Chloroquine dose (mg/day) ^a^	150 (100–200)	-	-
Hydroxychloroquine % (*n*) ^b^	30.5 (54/177)	-	-
Hydroxychloroquine dose (mg/day) ^a^	200 (150–200)		
Antihypertensives % (*n*) ^b^	32 (19/60)	-	-

^a^ Data shown as median (percentile: p5th–p95th), *p* value: U Mann–Whitney test. ^b^ Data shown as percentages (*n*), ^c^ Data shown as mean and standard deviation, *p* value: Student’s *t*-test. The bold numbers indicate variables with significant differences. **SLE:** systemic lupus erythematosus patients; **CS:** control subjects; **BMI:** body mass index; **WHR:** waist to hip ratio; **(WHtR)**; **WHtR:** waist to height ratio (cm/cm); **HDL-C:** high-density lipoprotein cholesterol; **LDL-C:** low-density lipoprotein cholesterol; **TC:** total cholesterol; **CMI:** cardiometabolic index; CMI = (triglycerides/HDL-C); **LAP:** lipid accumulation products; LAP = (waist in cm − 58)*(triglycerides mmol/L).

**Table 2:** Biochemical and cardiometabolic status stratified according to CVD risk by CRP in SLE patients and CS.

Variable	CVD Risk by CRP in SLE Patients			*p* Value	CVD Risk by CRP in CS			*p* Value
	Low Risk (<1 mg/L) *n* = 30	Average Risk (≥1 to <3 mg/L) *n* = 33	High Risk (≥3 mg/L) *n* = 113		Low Risk (<1 mg/L) *n* = 84	Average Risk (≥1 to <3 mg/L) *n* = 46	High Risk (≥3 mg/L) *n* = 45	
Glucose (mg/L) ^a^	85.9 (76.1–154)	86 (65–116)	89 (70–133)	0.71	84.9 (72.9–98.9)	86 (75.1–105.1)	96.1 (82.2–174.8)	**0.001**
Triglycerides (mg/L) ^a^	112 (45–214)	83 (47–287)	124 (61–245.7)	**<0.001**	65.6 (36–135.3)	76.1 (45.8–170.1)	109.3 (49.7–225.2)	**<0.001**
Total cholesterol (mg/L) ^a^	163 (110.7–251)	159 (109–260)	171 (119.5–249.5)	0.25	162.7 (123.6–228.6)	169.2 (118.8–229.9)	190 (131.7–274)	**0.01**
LDL-C (mg/L) ^a^	84.7 (53.2–128.7)	89.5 (53.6–172)	72.3 (41.3–138.1)	**0.03**	92 (59–157.2)	96 (55–147.2)	110.7 (63.1–180.2)	**<0.01**
HDL-C (mg/L) ^a^	41.3 (19.1–71.6)	45.4 (21.7–71.2)	26.2 (12.8–62.7)	**<0.001**	54.6 (40.3–83)	50.4 (32.6–70.6)	45.5 (31.7–66.4)	**<0.001**
Castelli index (TC/HDL-C) ^a^	3.7 (2.4–10.8)	3.7 (2.2–8.4)	6.5 (2.8–14.9)	**<0.001**	2.8 (2.1–4.9)	3.2 (2.4–6.5)	4.3 (2.3–6.3)	**<0.001**
**Castelli CVD risk *%* (*n*) ^b^**								
Low risk (<4.5)	78.6 (22/28)	70 (23/33)	36 (37/104)		90.5 (76/84)	73.9 (34/46)	53.3 (24/45)	
Moderate risk (≥4.5 to <7.0)	10.7 (3/28)	24 (8/33)	16.3 (17/104)	**<0.001**	8.3 (7/84)	21.7(10/46)	46.7 (21/45)	**<0.001**
High risk (≥7.0)	10.7 (3/28)	6 (2/33)	48 (50/104)		1.2 (1/84)	4.4 (2/46)	0 (0/45)	
Kannel Index (LDL-C/HDL-C) ^a^	1.8 (1–4.4)	2 (1.1–3.4)	2.5 (1.2–6.3)	**<0.001**	1.7 (0.86–3.2)	1.8 (1.1–3.5)	2.5 (1.2–3.8)	**<0.001**
**Kannel CVD risk % (*n*) ^b^**								
Low risk (<3)	82.1 (23/28)	87.9 (29/33)	59.6 (62/104)	**0.001**	93 (78/84)	84.4 (38/45)	66.7 (30/45)	**0.001**
High risk (≥3)	17.9 (5/28)	12.1 (4/33)	40.4 (42/104)		7 (6/84)	15.6 (7/45)	33.3 (15/45)	
Triglycerides/HDL-C ratio	1.9 (1.1–9.3)	2.1 (0.8–5.6)	4.4 (1.3–15)	**<0.001**	1.2 (0.4–2.8)	1.4 (0.7–5)	2.5 (0.8–5.8)	**<0.001**
**Triglycerides/HDL-C risk % (*n*) ^b^**								
Low risk (<3)	68 (19/28)	76 (25/33)	29 (30/104)	**<0.001**	96.4 (81/84)	80.4 (37/46)	58 (26/45)	**<0.001**
High risk (≥3)	32 (9/28)	24 (8/33)	71 (74/104)		3.6 (3/84)	19.6 (9/46)	42 (19/45)	
LAP (score) ^a^	25 (4.2–72.5)	26.1 (7.7–86)	41.7 (7.6–95.2)	**0.02**	8.9 (1.5–38.3)	18.9 (4.4–59.7)	41.8 (5.5–106.2)	**<0.001**
CMI (score) ^a^	1 (0.4–3.5)	1.1 (0.4–3)	1.4 (0.4–3.3)	**0.03**	0.5 (0.19–2.4)	0.8 (0.3–2.9)	1.2 (0.4–10)	**<0.001**

^a^ Data provided in median (percentile: p5th–p95th), *p* value: Kruskal–Wallis test. ^b^ Data provided in percentages (*n*), *p* value: Pearson χ^2^ test. The bold numbers indicate variables with significant differences. **SLE:** systemic lupus erythematosus patients; **CS:** control subjects; **LDL-C:** low-density lipoprotein cholesterol; **HDL-C:** high-density lipoprotein cholesterol; **CVD:** cardiovascular disease; **TC:** total cholesterol; **LAP:** lipid accumulation products; LAP = (waist in cm − 58)*(triglycerides mmol/L); **CMI:** cardiometabolic index; CMI = (triglycerides/HDL-C).

**Table 3:** Correlations between body composition, cardiometabolic status, and SLE clinical features with CRP in both study groups.

Variables	All Participants		SLE Patients		CS	
	CRP (mg/L)		CRP (mg/L)		CRP (mg/L)	
	* r	*p* Value	* r	*p* Value	* r	*p* Value
**Body composition**						
Weight (kg)	0.44	**<0.001**	0.22	**<0.01**	0.56	**<0.001**
Waist (cm)	0.52	**<0.001**	0.19	0.05	0.60	**<0.001**
BMI (kg/m^2^)	0.52	**<0.001**	0.28	**<0.001**	0.63	**<0.001**
WHR (score)	0.42	**<0.001**	0.11	0.24	0.45	**<0.001**
WHtR (score)	0.53	**<0.001**	0.21	**0.03**	0.59	**<0.001**
Fat mass (%)	0.50	**<0.001**	0.21	**0.03**	0.65	**<0.001**
Muscle mass (kg)	−0.48	**<0.001**	−0.17	0.08	−0.61	**<0.001**
Body water (%)	−0.51	**<0.001**	−0.17	0.08	−0.65	**<0.001**
**Cardiometabolic status**						
Glucose (mg/L)	0.22	**<0.001**	0.08	0.29	0.39	**<0.001**
Triglycerides (mg/L)	0.42	**<0.001**	0.18	**0.01**	0.42	**<0.001**
Total cholesterol (mg/L)	0.07	0.15	0.01	0.84	0.20	**<0.01**
LDL-C (mg/L)	−0.13	**0.01**	−0.24	**0.001**	0.21	**<0.01**
HDL-C (mg/L)	−0.44	**<0.001**	−0.37	**<0.001**	−0.31	**<0.001**
Castelli index (TC/HDL-C)	0.47	**<0.001**	0.35	**<0.001**	0.40	**<0.001**
Kannel Index (LDL-C/HDL-C)	0.36	**<0.001**	0.23	**<0.01**	0.36	**<0.001**
Triglycerides/HDL-C ratio (score)	0.52	**<0.001**	0.38	**<0.001**	0.45	**<0.001**
LAP (score)	0.51	**<0.001**	0.18	0.06	0.59	**<0.001**
CMI (score)	0.46	**<0.001**	0.21	**0.03**	0.51	**<0.001**
**SLE clinical features**						
Mex-SLEDAI (score)	-	-	0.22	**<0.01**	-	-
Disease duration (years)	-	-	0.20	**<0.01**	-	-

* Spearman correlation test. The bold numbers indicate variables with significant differences. **SLE:** systemic lupus erythematosus; **CS:** control subjects; **CRP:** C-reactive protein; **BMI:** body mass index; **WHR:** waist to hip ratio; **WHtR**: waist to height ratio (cm/cm); **LDL-C:** low-density lipoprotein cholesterol; **HDL-C:** high-density lipoprotein cholesterol; **TC:** total cholesterol; **LAP:** lipid accumulation products; LAP = (waist in cm − 58)*(triglycerides mmol/L); **CMI:** cardiometabolic index; CMI = (triglycerides/HDL-C).

## Data Availability

Data used to support the findings of this study are available from the corresponding author upon reasonable request.
